# Efficacy and Safety of Bendamustine-Rituximab as Frontline Therapy for Indolent Non-Hodgkin Lymphoma: A Real-World, Single-Center, Retrospective Study

**DOI:** 10.7759/cureus.66124

**Published:** 2024-08-04

**Authors:** Tevy Chan, Jean-Nicolas Champagne, Jean-Samuel Boudreault

**Affiliations:** 1 Geriatric Medicine, McGill University, Montreal, CAN; 2 Hematology and Medical Oncology, Hopital du Sacre-Coeur de Montréal, Montreal, CAN

**Keywords:** treatment toxicities, treatment efficacy, frontline therapy, bendamustine, indolent lymphoma

## Abstract

Background

The use of bendamustine with an anti-CD20 monoclonal antibody as frontline therapy for indolent non-Hodgkin lymphoma (NHL) has become a standard of care. We aimed to evaluate the real-world efficacy and safety of bendamustine-rituximab (BR) frontline therapy for indolent NHL.

Patients and methods

Patients with indolent NHL treated with frontline BR therapy in Hôpital du Sacré-Coeur de Montréal, from January 2015 to August 2018 were included in this retrospective study.

Results

Our cohort included 42 adults with a median age of 63 years. Follicular lymphoma was the most common histology (n = 31, 74%). Most patients had advanced disease (Lugano stage III or IV, 88%). The overall response rate was 84% (complete response = 62% and partial response = 22%). Median progression-free survival (PFS) was not reached. At 30 months, PFS was 74.8% and overall survival was 90%. Grade 3-4 neutropenia occurred in 21% of patients. Infection-related adverse events were observed in 17 patients (40%). Most were grade 1 and 2 events (84%). One case of grade 5 progressive multifocal leukoencephalopathy related to John Cunningham (JC) virus reactivation was observed. The most common non-infectious-related adverse events were mild nausea and fatigue.

Conclusions

The efficacy and safety of BR treatment for indolent NHL were comparable in our real-life cohort compared to prior studies. This supports BR as a standard of care for indolent NHL. Future studies should assess whether the use of granulocyte-colony stimulating factors as primary prophylaxis effectively mitigates the hematological and infection-related adverse events related to BR therapy.

## Introduction

Indolent non-Hodgkin lymphomas (NHLs) are a heterogeneous group of malignancies including follicular lymphoma (FL), small lymphocytic lymphoma (SLL), certain cases of mantle cell lymphoma (MCL), marginal zone lymphoma (MZL), and lymphoplasmacytic lymphoma (LPL). These hematological disorders are characterized by their insidious disease course during which expectant management is often warranted [1]. Treatment is indicated when patients develop symptoms secondary to high disease burden or end-organ compromise. The GELF (Groupe d'Etude des Lymphomes Folliculaires) criteria are commonly used indications for treatment initiation and include any mass > 7 cm, involvement of ≥ three nodal sites each ≥ 3 cm, splenomegaly below the umbilical line, pleural or peritoneal effusion, cytopenias, and/or > 5.0 k/mcl circulating blood lymphoma cells [1,2]. Patients who do not meet these criteria are considered low-tumor burden and may be safely observed [2].

When indicated, recommended frontline treatment for indolent NHLs consists of a combination of immunotherapy (anti-CD20 monoclonal antibody rituximab or obinutuzumab), with a backbone of chemotherapy using bendamustine, CVP (cyclophosphamide, vincristine, and prednisone) or, occasionally, CHOP (cyclophosphamide, doxorubicin, vincristine, and prednisone) [1,3-5]. Bendamustine is a cytotoxic alkylating agent with proven efficacy in the treatment of indolent NHLs in several clinical trials.

Chemotherapy regimens R-CVP (rituximab plus cyclophosphamide, vincristine, and prednisone) and R-CHOP (rituximab plus cyclophosphamide, doxorubicin, vincristine, and prednisone) were commonly used until the publication of the StiL and BRIGHT trials, which favored the bendamustine-rituximab (BR) combination in the frontline [6,7]. The StiL randomized clinical trial showed bendamustine's superiority to CHOP in providing longer median progression-free survival (PFS) of 69.5 months for BR vs. 31.2 months for R-CHOP, as well as a low toxicity profile [6]. In the BRIGHT trial, BR was found to be non-inferior to R-CHOP or R-CVP [7]. Additionally, in the recent GALLIUM study, a second-generation anti-CD20, obinutuzumab, was studied for frontline treatment in combination with a chemotherapy backbone. A majority of patients received bendamustine backbone. Although this trial has shown improved PFS, high rates of grade 3 or higher infections were noted [8]. Overall, an anti-CD20, in combination with bendamustine backbone is now the preferred frontline therapy for most patients due to its efficacy and lower toxicity profile [1]. There is also an interest in using anti-CD20 monoclonal antibodies as maintenance therapy after induction. Maintenance rituximab after R-CVP or R-CHOP has been shown to improve PFS in the PRIMA trial, but no change in improved overall survival (OS) was demonstrated with longer follow-up [9,10]. Only limited data exist on the safety and efficacy of rituximab maintenance after bendamustine. The GALLIUM study has included maintenance obinutuzumab for their experimental arm after bendamustine-obinutuzumab induction therapy, but maintenance therapy was not randomized [8]. In fact, a concern for increased toxicity was raised early, and fatal cardiac events were reported; however, a retrospective study demonstrated the relative safety of rituximab maintenance after bendamustine treatment [11]. Unfortunately, to date, no phase III trial has demonstrated a clear PFS or OS benefit of anti-CD20 maintenance after bendamustine backbone therapy. Additionally, increased susceptibility to infection was of great concern, especially during the COVID-19 pandemic. Given the paucity of data, this practice is variable in different centers, and maintenance rituximab after bendamustine treatment is not a common practice in our center.

At Hôpital du Sacré-Cœur de Montréal, the frontline BR regimen was adopted as part of our frontline protocol for patients affected by indolent NHL soon after the publication of the results from the StiL and BRIGHT clinical trials. In this retrospective study, we aim to evaluate the real-world efficacy and safety of BR frontline therapy for indolent NHL.

## Materials and methods

Patients and baseline characteristics

Patients included in this study were adults who received BR as frontline therapy for indolent NHL at our center from January 2015 to August 2018. Included in histology were FL (grade 1-3A), MZL, and LPL based on the World Health Organization (WHO) classification. Patients were identified by the hospital archives list.

For FL patients, the Follicular Lymphoma International Prognostic Index (FLIPI) was retrospectively determined using data extracted from the chart, which included patients' age, Eastern Cooperative Oncology Group (ECOG) performance status, lymphoma staging according to the Lugano classification, number of involved nodal areas, hemoglobin level, and serum lactate dehydrogenase level. The presence or absence of constitutional symptoms and beta2-microglobulin (ß2-microglobulin) level were also collected from charts when available. Bone marrow involvement was determined from the results of available positron-emission tomography (PET) or bone marrow biopsy.

Informed consent was not deemed necessary for this retrospective chart review. Data were analyzed anonymously to maintain patient confidentiality. The study was approved by the Research Ethics Committee of the Centre intégré universitaire de santé et de services sociaux du Nord-de-l’Île-de-Montréal (CIUSSS du NIM).

Treatment

As per the institution's protocol, patients received rituximab at the dose of 375 mg/m^2^ on day one of each cycle. On days one and two, patients received an intravenous infusion of bendamustine at a standard dose of 90 mg/m^2^ or at a reduced dose of 60 mg/m^2^. Treatment was given every four weeks and continued for six cycles unless disease progression, histology transformation, unacceptable toxicity, or the patient's refusal to pursue treatment.

Treatment response and toxicity assessment

Treatment response was assessed based on pre- and post-treatment PET or computed tomography (CT) scan results and was classified according to the Lugano criteria for response assessment in lymphoma [12].

Medical records were used to retrieve information relative to treatment toxicity and adverse events. Toxicities were graded according to the National Cancer Institute Common Terminology Criteria for Adverse Events (CTCAE) version 5.0 [13]. Adverse events were defined as occurring after BR initiation up to 100 days post treatment. Data from the pharmacy department and the hospital's blood bank (Traceline) were used to determine the prescription of granulocyte-colony stimulating factor (G-CSF) and the need for red blood cell (RBC) or platelet transfusion during treatment.

Statistical analysis

Analysis was performed in March 2020. Descriptive statistics were used to describe patient characteristics, treatment response, and occurrence of adverse events. PFS was obtained using the Kaplan-Meier method and was defined as the time from initiation of the BR to the date of first documented progression, death from any cause, or censoring at the last follow-up, which was October 20th, 2019.

## Results

Patient characteristics

In total, 42 adults were treated with frontline therapy BR for indolent NHL at our institution up to August 2018. Their median age was 63 (Table 1). Follicular lymphoma was the most common histology (FL (74%) vs. MZL (21%) and LPL (5%)). Most patients had advanced disease (Lugano stage III or IV (88%) vs. stage I-II (12%)). FLIPI intermediate- and high-risk prognostic groups accounted for most cases, i.e., 38% and 50%, respectively. Of the patients, 86% had good performance status (ECOG 0-1). Constitutional symptoms were noted in 31% of patients as per charts, and bone marrow involvement in 48% of cases. In 17% of cases, bone marrow involvement status was unknown as bone marrow biopsy was not systematically performed. It was also noted that ß2-microglobulin level was not routinely requested before 2018.

**Table 1:** Patient characteristics. ECOG: Eastern Cooperative Oncology Group; LDH: lactate dehydrogenase; FL: follicular lymphoma; FLIPI: Follicular Lymphoma International Prognostic Index.

	Number of patients (%)
Age, median, years (range)	62.5 (34-82)
Sex (male/female)	23/19
Baseline ECOG performance status	
0-1	36 (86%)
2	5 (12%)
3	1 (2%)
4	0
Histologic classification	
Marginal zone	9 (21%)
Follicular, grade 1	13 (31%)
Follicular, grade 2	13 (31%)
Follicular, grade 3a	2 (5%)
Follicular, grade unknown	3 (7%)
Lymphoplasmacytic lymphoma	2 (5%)
Lugano classification	
Stage I	2 (5%)
Stage II	3 (7%)
Stage III	12 (29%)
Stage IV	25 (60%)
B symptoms	13 (31%)
Bone marrow involvement	
Present	20 (48%)
Absent	15 (36%)
Unknown	7 (17%)
Extranodal involved sites ≥1	14 (33%)
LDH	
High	12 (29%)
Normal	27 (64%)
Unknown	3 (7%)
B2-microglobulin	
High	5 (12%)
Normal	7 (17%)
Unknown	30 (71%)
FL prognostic groups according to the FLIPI (total number of FL = 31)	
Low risk (0-1 risk factor)	5 (16%)
Intermediate risk (2 risk factors)	10 (32%)
High risk (3-5 risk factors)	16 (52%)

The completion rate of the six cycles of BR was 79% (n = 33) for the planned six cycles of BR. Only one patient received an upfront reduced dose (60 mg/m^2^) and completed six cycles. Among those who discontinued BR (total n = 9), the main reason for discontinuation was due to toxicities (n = 7, 78%). Histology transformation leading to treatment cessation occurred in two patients (22%).

Treatment response

**Figure 1:**
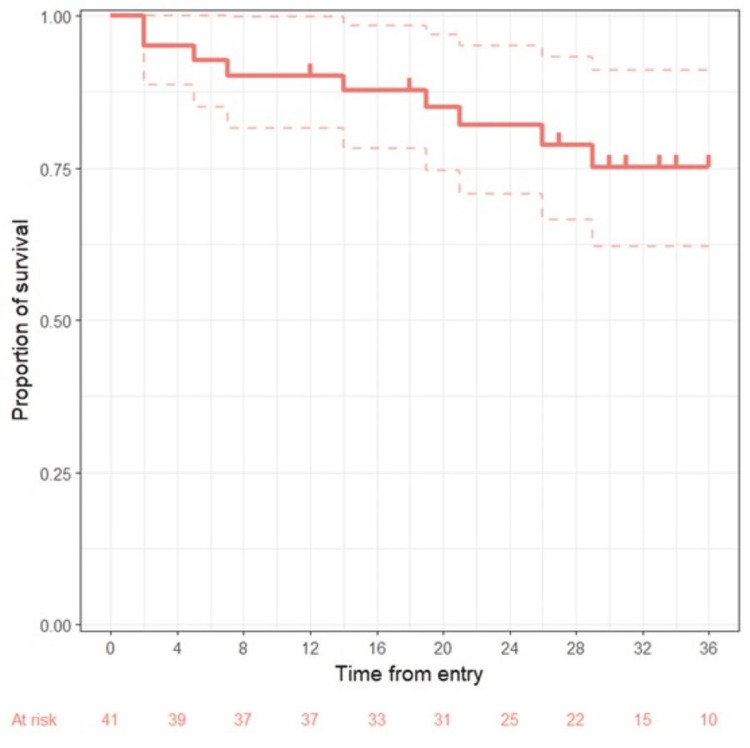
Progression-free survival time. Progression-free survival in months. 95% confidence intervals are represented with dashed lines.

In our patient cohort, the overall response rate (ORR) was 84%, with 62% of the patients achieving complete response, and 22% partial response (Table 2). Assessment of treatment response was not possible in five patients, due to one patient lost to follow-up, three patients with adverse events requiring treatment discontinuation and did not have imaging to evaluate response after regimen cessation, and one patient with grade 5 adverse event. Figure 1 shows progression-free survival time in months with 95% confidence intervals. Estimation of the overall survival was not possible due to the small number of samples.

**Table 2:** Treatment response. CR: complete response; PR: partial response; SD: stable disease; PD: progressive disease.

	CR	PR	SD	PD	Total
Number of patients	23	8	3	3	37
Percentage	62%	22%	8%	8%	100%

Two patients had histology transformation, both into diffuse large B cell lymphoma (DLBCL). One of them occurred after two cycles of the bendamustine-rituximab therapy, whereas the other one occurred three months after the completion of the planned six cycles of BR. Both patients subsequently received R-CHOP for their disease transformation, but both were deceased. Median PFS was not reached. With a median follow-up of 29.2 months, PFS was 74.8% at 30 months and OS was 90%, excluding one patient lost to follow-up (Figure 1).

Adverse events

**Figure 2:**
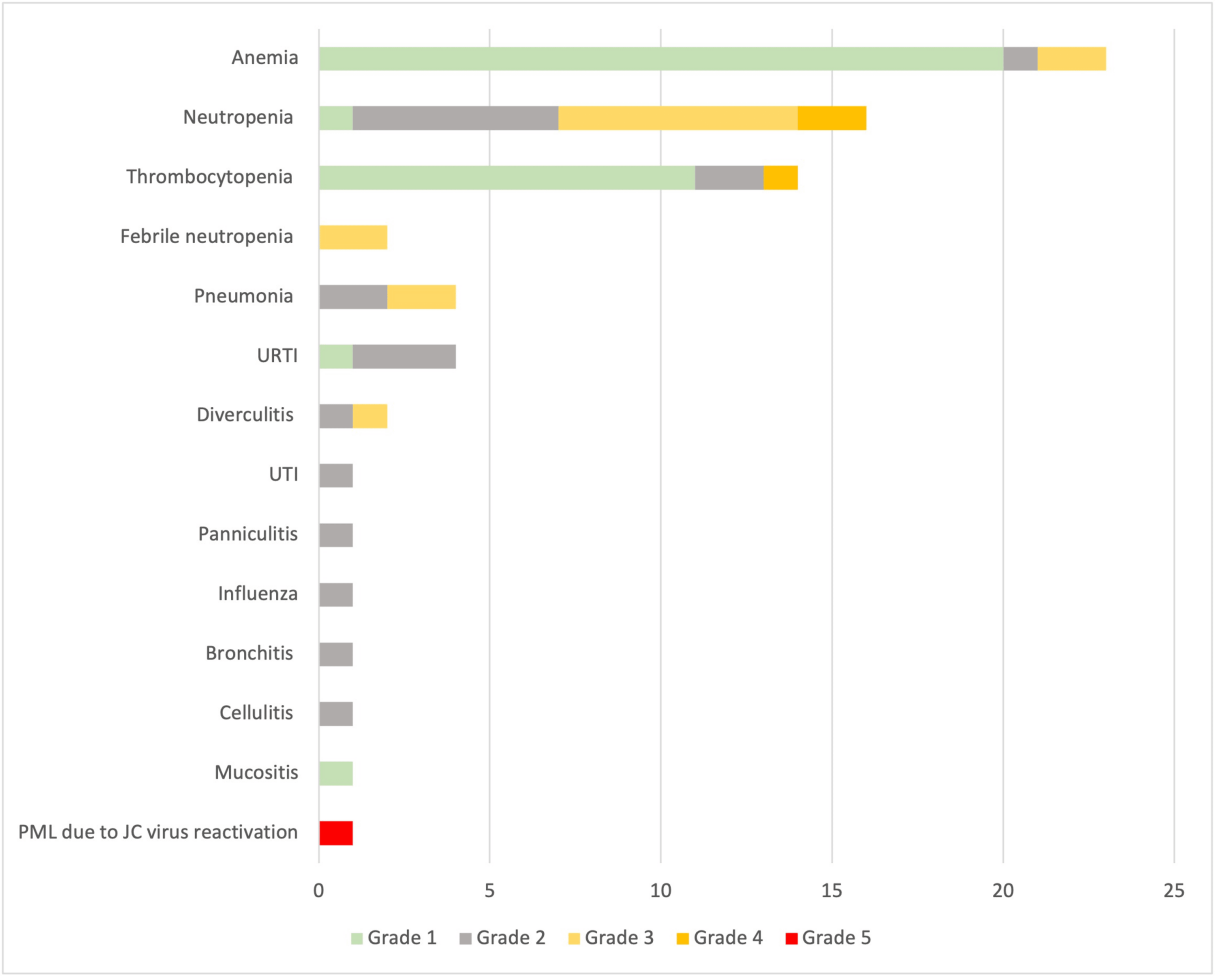
Hematologic and infection-related adverse events in patients receiving at least one dose of bendamustine-rituximab. JC virus: John Cunningham virus; PML: progressive multifocal leukoencephalopathy; URTI: upper respiratory tract infection; UTI: urinary tract infection.

The most common grade 3-4 hematological adverse event related to bendamustine-rituximab therapy in our cohort was neutropenia, occurring in 21% of patients (Table 3 and Figure 2). Grade 3-4 anemia, thrombocytopenia, and febrile neutropenia only occurred in a minority of cases (≤ 5%). G-CSF was administered to 10 patients.

**Table 3:** Hematological adverse events in patients receiving at least one dose of bendamustine-rituximab.

Number of patients (%)	Grade 1	Grade 2	Grade 3	Grade 4	Grade 3-4
Neutropenia	1 (2%)	6 (14%)	7 (17%)	2 (5%)	9 (21%)
Anemia	20 (48%)	1 (2%)	2 (5%)	0	2 (5%)
Thrombocytopenia	11 (26%)	2 (5%)	0	1 (2%)	1 (2%)
Febrile neutropenia	0	0	2 (5%)	0	2 (5%)

Infection-related adverse events were observed in 17 patients (40% of cohort). Most were of mild-to-moderate severity (21 grade 1 and 2 events, 84%) (Table 4 and Figure 2). There was, however, one grade 5 adverse event observed. A patient developed ataxia, gait impairment, falls, and dysarthria following the 5th cycle of bendamustine-rituximab therapy. Brain imaging showed white matter changes in the cerebellum and lumbar puncture revealed a John Cunningham (JC) virus infection. The patient’s condition rapidly declined due to progressive multifocal leukoencephalopathy (PML) related to JC virus reactivation and he was transferred to hospice care, where he deceased.

**Table 4:** Infection-related adverse events in patients receiving at least one dose of bendamustine-rituximab. JC virus: John Cunningham virus; PML: progressive multifocal leukoencephalopathy; URTI: upper respiratory tract infection; UTI: urinary tract infection.

Number of patients	Grade 1	Grade 2	Grade 3	Grade 4	Grade 5
Bronchitis		1			
Cellulitis		1			
Diverticulitis		1	1		
Influenza		1			
Mucositis	1				
Panniculitis		1			
PML due to JC virus reactivation					1
Pneumonia		2	2		
URTI	1	3			
UTI		1			

**Figure 3:**
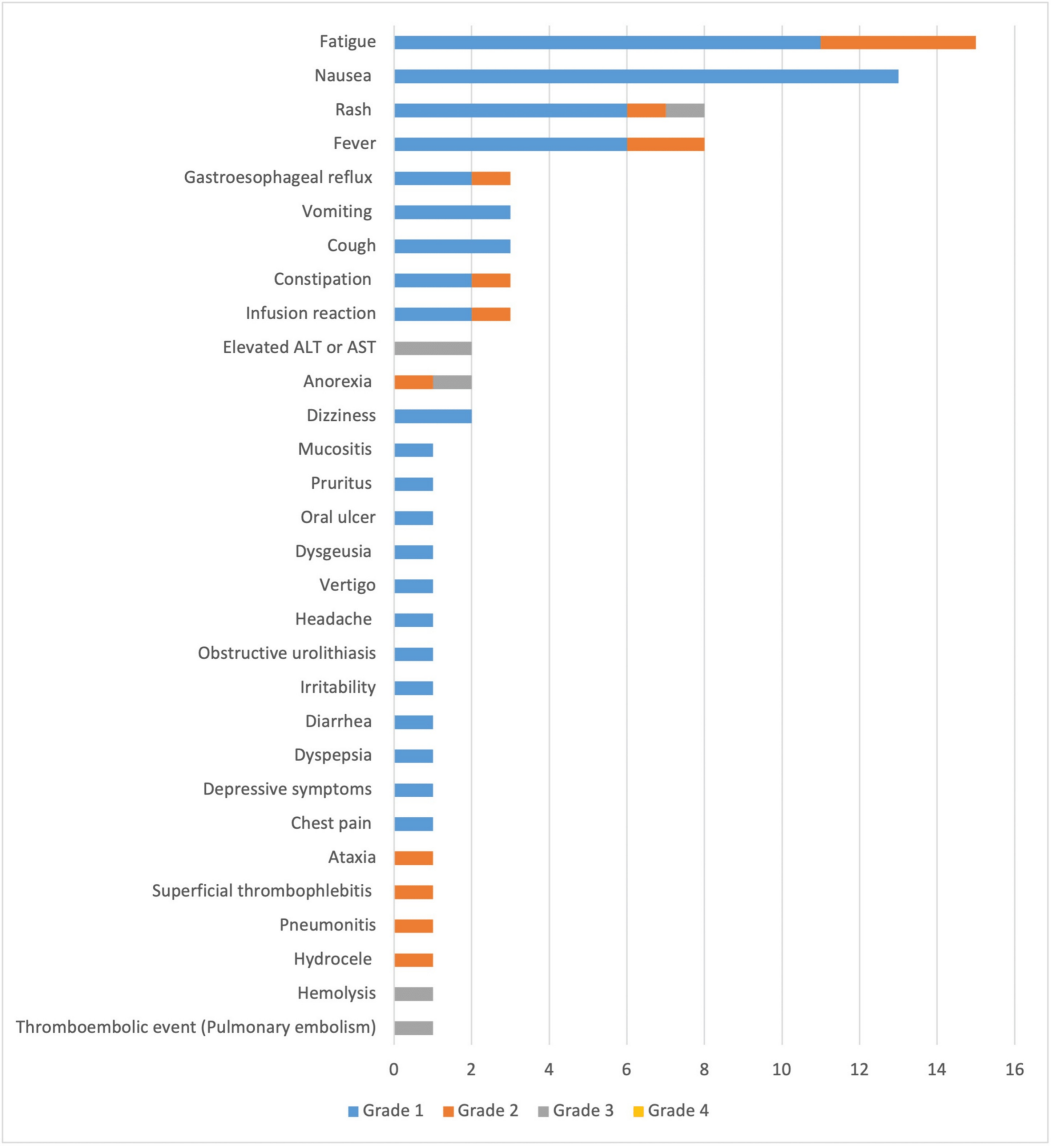
Non-infection-related adverse events in patients receiving at least one dose of bendamustine-rituximab. ALT: alanine transaminase; AST: aspartate transaminase.

The most common non-infectious related adverse events observed were mild nausea and fatigue. A detailed list of non-infection-related adverse events is available in Table 5 and Figure 3. Most of them were mild (grade 1-2). There was one case of suspected drug-induced pneumonitis requiring therapy discontinuation after three cycles.

**Table 5:** Non-infection-related adverse events in patients receiving at least one dose of bendamustine-rituximab. ALT: alanine transaminase; AST: aspartate transaminase.

Number of patients	Grade 1	Grade 2	Grade 3	Grade 4	Grade 5
Anorexia		1	1		
Ataxia		1			
Chest pain	1				
Constipation	2	1			
Cough	3				
Depressive symptoms	1				
Diarrhea	1				
Dizziness	2				
Dysgeusia	1				
Dyspepsia	1				
Elevated ALT			2		
Elevated AST			2		
Fatigue	11	4			
Fever	6	2			
Gastroesophageal reflux	2	1			
Headache	1				
Hemolysis			1		
Hydrocele		1			
Infusion reaction	2	1			
Irritability	1				
Nausea	13				
Obstructive urolithiasis	1				
Oral ulcer	1				
Pneumonitis		1			
Pruritus	1				
Pulmonary embolism			1		
Rash	6	1	1		
Superficial thrombophlebitis		1			
Vertigo	1				
Vomiting	3				

## Discussion

After the publication of the StiL and BRIGHT trials, the BR combination became an increasingly used first-line option for the treatment of indolent NHL [6,7], and this approach was rapidly adopted in our center. The results from our real-life study are comparable to those of the randomized trials that have set BR to be a favored frontline treatment. In our cohort, the PFS after BR was 74.8% at 30 months and demonstrated an acceptable tolerability profile.

Treatment efficacy

In our cohort, the ORR was 83.7% compared to the ORR of 92.7% and 96.7% reported in StiL and BRIGHT trials, respectively. Although these numbers are slightly lower, PFS seems to be comparable to what is reported in the literature. With our short follow-up of 29.2 months, this regimen seems to be as effective as previously described in StiL and BRIGHT trials, with only a few patients demonstrating progression. Early progressors also seem to be comparable to what is reported in the literature of approximately 20% [14], even with our small number of patients. Additional follow-up may be warranted to compare our long-term results with what is reported in the literature.

Despite the PRIMA trial demonstrating improved PFS from two-year rituximab maintenance, no patient received bendamustine prior to randomization [9]. Moreover, there was a signal toward increased risk of fatal events in patients over 70 years old receiving maintenance therapy in the GALLIUM trial [8]. However, a retrospective study including 640 patients with FL demonstrated a PFS benefit in patients achieving partial remission following BR induction, but not for patients in complete remission. Fortunately, the risk of fatal events was low and comparable to those who received no maintenance [11]. Clinicians who choose to administer anti-CD20 maintenance should stop after two years, because increased toxicity has been shown, with limited additional benefit. Altogether, given the paucity of data at that time, it was not the current standard of practice to provide patients with rituximab maintenance after frontline BR. As such, no patient with follicular lymphoma received maintenance in our cohort.

Tolerability of treatment

First off, it is worth mentioning that nine patients (21.4%) did not complete the planned six cycles of treatment, the majority of which (77.8%) were due to adverse events and tolerability of the treatment. In the follicular lymphoma group, patients who did not complete the planned six cycles because of toxicity, PFS was 80% at 24 months, compared to 86.4%. No statistical difference was noted between those two groups, but these data should not be extrapolated, as very few patients were included in these subgroups.

Some authors propose that patients over 70 years old should be offered adjusted bendamustine doses to mitigate side effects from chemotherapy [15,16]. However, in our cohort, which comprised 11 patients (26%) who were aged 70 years or over, only one patient received a 60 mg/m2, and two others received a 5-10% reduction in dose.

We perceived the treatment as being well tolerated in the younger population, but tolerance may be difficult in patients over 70 years old [16]. A real-world study of BR treatment of indolent NHL in older patients showed that BR efficacy was maintained even in very old patients (≥80 years), though dose reduction was common. Alternative chemotherapy backbone with fewer toxicities (RCOP (rituximab, cyclophosphamide, vincristine, and prednisone) or single-agent rituximab) is an acceptable alternative in elderly patients or with major comorbid conditions.

In our cohort, grade 3-4 neutropenia was reported in 21%. Despite this high number, G-CSF use in our cohort was erratic, seen in only 10 patients. In addition to neutropenia, infectious complications were observed in as many as 17 patients (40%). A patient developed grade 5 JC virus reactivation leading to PML to which he succumbed. Other studies also showed that myelosuppression is the most common grade 3-4 adverse event associated with the bendamustine regimen [17]. Given these adverse events, the findings encourage strong consideration for prophylactic G-CSF as primary prevention in most patients. In fact, prospective studies have shown that the use of G-CSF as primary prophylaxis was associated with reduced incidence of chemotherapy disruptions due to febrile neutropenia (FN), as well as the days of hospitalization related to FN [18].

Another safety concern for the increased use of BR in the frontline is the higher incidence of secondary neoplasm [19]. Updated results from the BRIGHT trial showed that 21.7% of malignancy was reported in the BR group compared to 12.1% in the R-CHOP/R-CVP group [20]. Although our cohort only had a shorter follow-up, three patients had a new cancer diagnosis following BR treatment. These were early-stage lung cancer in two patients, and liposarcoma in another.

BR for all?

Given the results from previously mentioned trials, BR has appropriately been favored as a first-line treatment in most patients. However, given its fair number of adverse events (stated previously), BR may not be the best option for all. Many experts wrote reviews on the subject, and consideration for alternative regimens should be made and tailored according to the patients [1,3,15,21,22].

Other frontline therapies should also be considered, such as the rituximab-lenalidomide (R2), supported by the RELEVANCE trial [23]. This study demonstrated non-inferiority in terms of PFS compared to rituximab chemotherapy and decreased adverse events in terms of neutropenia and infections. Overall survival was high and similar in both groups. Moreover, for a patient who presents with multiple comorbidities or whose fitness may preclude chemotherapy treatment, rituximab monotherapy may also be considered [24]. Although response rates and PFS are low, these treatments may be beneficial for a subset of unfit patients who would not be able to tolerate chemotherapy otherwise. Additionally, long-term follow-up has shown that up to 30% of patients did not require additional therapy 10 years after rituximab monotherapy [25]. This may be reasonable for some patients.

Another consideration, although not reflected in this study, is treatment sequencing [26]. Younger patients afflicted with indolent lymphomas seem to express shorter survival compared to age-matched control, as such a vast majority of them may experience multiple relapses, requiring multiple treatments throughout their lifespan compared to older patients [27]. Similarly, patients with high-risk disease may experience multiple relapses as well. To prevent lymphoma-related death in this population, new treatment strategies are being explored. Currently, there is increasing evidence for the use of chimeric antigen receptor T-cell (CAR-T) for relapsed/refractory indolent NHL [28,29]. However, it has been proposed that prior bendamustine exposure may alter T-cell kinetics, therefore negatively impacting the clinical outcomes of CAR-T in aggressive lymphoma [30]. This area of uncertainty is under investigation, but if bendamustine may alter ulterior treatment, consideration for alternative regimens may be sought.

## Conclusions

The safety and efficacy of BR treatment for indolent lymphoma are similar in our real-life cohort of patients compared to prior studies. Additional follow-up is warranted to determine long-term PFS and OS in our cohort. Future studies should assess whether the use of G-CSF as primary prophylaxis effectively mitigates the hematological and infectious adverse events related to BR therapy.
